# The relationship between emotional self-awareness, emotion regulation, and diabetes distress among Italian and Dutch adults with type 1 diabetes

**DOI:** 10.3389/fpsyg.2023.1288550

**Published:** 2023-11-22

**Authors:** Giulia Bassi, Jiska Embaye, Maartje de Wit, Frank J. Snoek, Silvia Salcuni

**Affiliations:** ^1^Department of Developmental and Socialization Psychology, University of Padova, Padua, Italy; ^2^Department of Medical Psychology, Amsterdam UMC Location VUmc, VU University Amsterdam, Amsterdam, Netherlands; ^3^Amsterdam Public Health, Mental Health, Amsterdam, Netherlands

**Keywords:** emotional self-awareness, emotion regulation, diabetes distress, type 1 diabetes, adults, cross-sectional study

## Abstract

**Objective:**

Evidence suggests that many adults with type 1 diabetes (T1D) experience clinically relevant levels of diabetes distress, indicating coping difficulties. Studies have primarily focused on emotion regulation as a possible construct to be addressed in psychological interventions to alleviate diabetes distress. This study extends the literature by investigating the cross-sectional association between emotion regulation, diabetes distress and the construct of emotional self-awareness as an additional variable to be considered in potentially reducing diabetes distress.

**Methods:**

Via an online survey, data was collected on emotional self-awareness dimensions (attention to feelings, clarity of feelings), emotion regulation strategies (cognitive reappraisal, expressive suppression, mood repair) and diabetes distress, along with self-reported clinical and sociodemographic information. Multiple linear regression with stepwise backward method was used to examine associations, controlling for country.

**Results:**

*N* = 262 Italian and Dutch adults with T1D (80.5% women, *M* = 38.12 years, SD = 12.14) participated. Clarity of feelings was significantly negatively associated with diabetes distress, resulting in a medium effect size (*β* = −0.22, *p* < 0.001). Likewise, mood repair was negatively related to diabetes distress, showing a small effect size (*β* = −0.26, *p* < 0.001).

**Conclusion:**

These findings shed light on the importance of a dimension of emotional self-awareness, namely clarity of feelings. This represents the ability to identify one’s emotional states and discriminate between them. Thus, it should be considered in psychological interventions, such as mentalization-based treatment, that might contribute to alleviating T1D-related distress.

## 1 Introduction

Diabetes-related distress, or simply diabetes distress, refers to experiencing difficulties in regulating one’s emotions in response to the burden of living with and self-managing the diabetes (Polonsky et al., 1995). Studies have shown that diabetes distress is highly prevalent in adults with diabetes (Nicolucci et al., 2013) and is negatively correlated with self-management behaviors (e.g., following a healthy diet, regular physical activity, and adherence to prescribed medication), and, subsequently with poorer glycemic outcomes (Fisher et al., 2008, 2016). Traditional behavioral interventions developed for adults with diabetes, ranging from multidisciplinary psycho-education to psychological interventions, have demonstrated to decrease diabetes distress, showing a medium effect size (Schmidt et al., 2018). Notwithstanding this, the presence of diabetes distress continues to represent a relevant clinical problem (Fisher et al., 2016). In this regard, relatively little is known about the specific emotional mechanisms underlying elevated diabetes distress, which could be the target of clinical interventions. Maladaptive emotion regulation strategies have been found to be associated with chronic negative affectivity (Compare et al., 2014) and, indeed, adaptive emotion regulation is crucial for healthy adaptation, ranging from affective functioning to social relationships (Gross, 2001; Gross and Thompson, 2007). In the context of diabetes, Fisher and colleagues (Fisher et al., 2018) found that three emotion regulation strategies, such as non-judging of inner experience (i.e., acceptance of emotions), nonreactivity to inner experience (i.e., non-impulsive reactions to emotions), and emotional processing (i.e., engagement with emotions) are directly and negatively related to diabetes distress. More recently, Coccaro and colleagues (Coccaro et al., 2021) conducted a cross-sectional study in which effective emotion skills presented a significantly negative relationship with diabetes distress, reaching a medium effect size. The authors suggested that psychological interventions targeting ineffective emotion regulation, *per se*, may help to reduce diabetes distress. Notably, the authors adopted the term emotion skills to describe the process of ineffective emotion regulation, defined as difficulties in identifying, evaluating, and controlling the expression of emotions. Following the Extended Process Model of emotion regulation (EPM) (Gross, 2015), people enact a dynamic and circular process in the management of emotions, which results from the constant evaluation of what is perceived in both their inner and outer world, as well as the actions associated with that evaluation. Specifically, the process of intrinsic emotion regulation concerns the ability to adopt strategies to maintain, increase or decrease one or more dimensions of one’s emotional response as a function of personal and social goals (Gross, 2002; Gratz and Roemer, 2004). Two important regulation strategies considered in research and clinical practice are cognitive reappraisal and expressive suppression. The first indicates the “attempt to reinterpret an emotion-eliciting situation in a way that alters its meaning and modifies its emotional impact” (Lazarus and Alfert, 1964; Gross, 1998; Gross and Thompson, 2007), while the second, refers to the “attempt to inhibit or reduce ongoing emotional-expressive behavior” (Gross, 1998; Gross and Thompson, 2007). Thus, cognitive reappraisal as an antecedent-focused strategy involves reflecting on how one is experiencing a situation that elicits an emotional response in order to subsequently develop and/or implement a coping strategy. This process implies being aware of one’s own emotions and corresponds to an adaptive emotion regulation strategy. In contrast, expressive suppression, as a response-focused strategy, is a mechanism that implies the non-capacity to authentically explore one’s emotional response to a given situation, resulting in a maladaptive emotion regulation strategy. Therefore, it does not necessarily imply being aware of one’s own emotions. Emotional self-awareness is one of the components of emotional intelligence and is composed of both the capacity of paying attention to emotions (i.e., attention to feelings) and of appropriately identifying emotions and discriminating between different emotional states (i.e., clarity of feelings). These are used to facilitate thinking and comprehend the meaning of emotions and their effects, as well as the capacity of managing them in order to appropriately adapt to social situations (i.e., mood repair) (Mayer and Salovey, 1997; Rieffe et al., 2008). Based on the theoretical framework of the present study, mood repair was considered within the emotional regulation process. Therefore, emotional self-awareness is interpreted as continuous reflective attention to one’s emotional experience and its underlying mechanisms, which can be learned and improved (Durlak et al., 2011). Emotional self-awareness constitutes a key element among various mechanisms that participate in regulating emotions (Gratz and Roemer, 2004; McMain et al., 2013), as it allows the use of the fundamental regulating strategies of modifying, accepting, and tolerating emotions (Gratz and Roemer, 2004; Berking et al., 2008; Lambie, 2008). The expression of emotions and problem-solving techniques are hindered by the difficulty of being aware of one’s own emotions, which leads to an increase in emotional distress (Derks et al., 2016). Baker and Berenbaum (2007) found that emotional self-awareness can be helpful in regulating emotions and/or adopting adaptive coping strategies. For example, people with higher levels of emotional self-awareness appear to be more likely to benefit from problem-focused coping strategies, whereas people with lower levels of emotional self-awareness may benefit more from emotion-focused coping strategies (Baker and Berenbaum, 2007). Both constructs of emotional self-awareness and emotion regulation are closely related to each other so that the act of regulating emotions is activated as a result of emotional self-awareness (Bagby and Taylor, 1997). Interestingly, emotional self-awareness has not yet been considered as a single construct that might contribute to the development of psychological interventions for adults with T1D, by possibly decreasing diabetes distress. To date, studies have shown that the overall construct of emotional intelligence presents a positive impact on diabetes self-management behaviors (Schinckus et al., 2018), and a negative association with HbA1c in adults with T1D (Zysberg et al., 2017), and that the implementation of diabetes self-care education significantly improved glycemic outcomes and emotional intelligence among adults with Type 2 Diabetes Mellitus (T2D) (Moghadam et al., 2018).

The present work, by means of a cross-sectional study design, explores the relationship between emotion regulation strategies, such as expressive suppression, cognitive reappraisal, and mood repair, the dimensions of emotional self-awareness, such as attention to feelings and clarity of feelings, and diabetes distress among Italian and Dutch adults with T1D. Our main research question was: *How do the constructs of emotional self-awareness* versus *emotion regulation contribute to explaining diabetes distress?* Mindful of the state-of-the-art outlined above, to our knowledge, this is the first study to address this relationship comparing different emotional processes in adults with T1D. The present investigation is expected to provide significant insights for both research and clinical practice, enabling the identification of relevant variables in the context of psychological interventions aimed at decreasing T1D-related distress.

## 2 Method

### 2.1 Recruitment and procedure

This study was part of a larger study on “Eating and Emotions,” a cross-sectional investigation among adults with T1D from the Netherlands and Italy (Embaye et al., 2023). Prior to recruitment, the sample size required to reach the set objective was calculated with the semPower package (Moshagen and Erdfelder, 2016) within the R environment, with a one-sided *p* < 0.05, a power of 0.80, and an RMSEA of 0.05. This resulted in a minimum sample size of *N* = 184 (*n* = 92 participants per country). Recruitment took place through sharing of an online survey on social networking groups of adults with T1D (e.g., Facebook, Instagram) and other platforms (e.g., WhatsApp, Gmail). The online survey was developed on two separate platforms, Survalyzer in the Netherlands and Google Forms in Italy. Before starting the survey, all participants received information about the study and gave their informed consent online anonymously. The study design was approved by both the ethics committee of the Amsterdam University Medical Centres-location VUmc (2021.0452) and the University of Padua, Italy (2021.4247) and was conducted according to the Declaration of Helsinki (Italian Law 196/2003, EU General Data Protection Regulation 679/2016).

### 2.2 Instruments

The McDonald’s omega (ω) was used in calculating the reliability of the instruments due to its capacity to provide robust estimates, especially in the context of multidimensional scales (Zinbarg et al., 2005; Dunn et al., 2014; Malkewitz et al., 2023).

#### 2.2.1 Diabetes distress

Diabetes distress was measured using the Problem Areas in Diabetes Scale-Short Form – 5 (PAID-SF-5) (McGuire et al., 2009). PAID-SF-5 is a unidimensional self-report measure, comprising 5 items evaluated on a 5-point Likert scale (0 “no problem” to 4 “severe problem”). Total scores range from 0 to 20, where higher scores indicate greater diabetes distress. A score equal to or higher than 8 suggests high diabetes-d distress. In the present study, the scale shows a McDonald’s ω equal to 0.84.

#### 2.2.2 Emotional self-awareness

The emotional self-awareness construct was assessed through the Trait Meta-Mood Scale (TMMS) (Salovey et al., 1995), a multidimensional self-report scale that measures the Perceived Emotional Intelligence construct, consisting of 30 items based on a 5-point Likert scale (from 1 “totally disagree” to 5 “totally agree”). The scale is characterized by a total score, which investigates the Perceived Emotional Intelligence, and by three subscales, (i) attention to feelings (14 items), which represents the capacity to perceive emotions, (ii) clarity of feelings (10 items), which indicates the capacity to identify and differentiate between different emotional states, and (iii) mood repair (6 items), which describes the capacity to regulate one’s emotional state so as to more effectively adapt to social situations. Based on the theoretical framework of the present study, clarity of feelings and attention to feelings were considered as components of the emotional self-awareness and mood repair within the emotional regulation process. In the present study, the McDonald’s ω for attention to feelings was 0.77, 0.81 for clarity of feelings, and 0.76 for mood repair.

#### 2.2.3 Emotion regulation strategies

The emotion regulation strategies were analyzed using the Emotion Regulation Questionnaire (ERQ) (Gross and John, 2003), which allows the identification of adaptive and maladaptive regulatory strategies of emotions. ERQ is a bi-factorial self-report measure, composed of 10 items based on a 7-point-Likert scale (from 1 “strongly disagree” to 7 “strongly agree”). The ERQ comprises two scales corresponding to two different emotion regulation strategies: (i) cognitive reappraisal (6 items) and (ii) expressive suppression (4 items). In the present study, the McDonald’s ω for cognitive reappraisal was 0.88, and for expressive suppression was 0.78.

The survey was designed also to capture all the relevant sociodemographic and clinical data, including gender identity, age, nationality, level of education, living situation, diabetes complications, mean glucose control over the past 3 months, and diabetes duration.

### 2.3 Data analysis

The data analyses were run using R custom code through the RStudio environment (R Core Team, 2019). Descriptive statistics (discrete and continuous variables) were computed in the overall sample and for both countries, separately. Pearson’s Chi-squared test (*χ*^2^) with Yates’ continuity correction was carried out to preliminarily investigate any differences in the discrete variables (i.e., diabetes complications, living situation, and education) between Italy and The Netherlands (*p* < 0.05). Cramer’s V effect size was calculated, in which a value of 0.1 represents a small effect size, 0.3 medium effect size, and 0.5 large effect size (Harald, 1946).

Likewise, an independent sample t-test was performed to explore any differences in the continuous variables [i.e., age, diabetes duration, mean glucose control, the three emotion regulation strategies (i.e., cognitive reappraisal, expressive suppression, and mood repair), the two dimensions of emotional self-awareness (i.e., clarity of feelings and attention to feelings), and diabetes distress] between the two countries (two-tailed *p* < 0.05). Effect sizes were evaluated using Cohen’s d, in which a value of 0.2 indicates a small effect size, 0.5 medium effect size, and 0.8 large effect size (Cohen, 1988). Notably, a value <7% refers to low glucose control while a value ≥7% indicates high glucose control.

Partial Pearson’s r correlation coefficients were computed to evaluate the associations between age, glucose control, diabetes distress, the three emotion regulation strategies, and the two dimensions of emotional self-awareness (*p* < 0.05), controlling for the diabetes duration in years, in both countries, separately.

Three multiple linear regression models were computed, by relying on the stepwise backward method in order to identify the statistical predictor(s) that might contribute to explaining diabetes distress. In the first model, the multiple predictor variables were the two dimensions of emotional self-awareness (clarity of feelings and attention to feelings), while in the second model, the predictor variables were the three emotion regulation strategies (cognitive reappraisal, expressive suppression and mood repair). Lastly, the third multiple linear model was run to understand the potential interaction effect between the results of the first and second models in diabetes distress. The effect sizes were evaluated using a rule-of-thumb for *f*^2^, with f^2^ ≥ 0.02 representing a small effect size, *f*^2^ ≥ 0.15 indicating a medium effect size, and *f*^2^ ≥ 0.35 a large effect size (Cohen et al., 2003). Country was included as a covariate in these analyses.

Of note, the terms predictors and influence are used to define the estimation of each independent variable’s contribution to the dependent one, without inferring the presence of a causal relationship between the variables.

## 3 Results

### 3.1 Sample characteristics and differences in discrete variables between countries

The characteristics of the samples corresponding to each country, as well as of the overall sample, are presented in Table 1. A total of *N* = 262 (*n* = 211, 80.5% cisgender female) adults with T1D aged 18–65 years (*mean* = 38.12, *SD* = 12.14) completed the entire survey. Most of them hold a university degree or an equivalent title, while they were evenly distributed with regard to living situation. Nevertheless, significant differences in living situations and the level of education emerged between the two countries, both showing a small effect size (*V* = 0.28, *V* = 0.18, respectively). Similarly, although the majority of the participants reported no diabetes-related complications, the results showed significant differences between the two countries, reporting a large effect size (*V* = 0.54). In particular, Dutch adults exhibit a higher prevalence of diabetes complications than Italian adults.

**Table 1:** Sample sociodemographic characteristics.

Sociodemographic Characteristics	Italy	The Netherlands	Total Sample	*χ* ^2^	*t* (df)	*p*
	*n* = 159	*n* = 103	*N* = 262			
Gender identity, *N* (%)				2.85		0.24
Cisgender female	125 (78.6)	86 (83.5)	211 (80.5)			
Cisgender male	34 (21.4)	16 (15.5)	50 (19.1)			
Non-binary	0 (0.0)	1 (1.0)	1 (0.4)			
Age, mean (SD)	38.87 (12.04)	36.36 (12.0)	38.12 (12.14)		1.65 (260)	0.51
Diabetes complications, *N* (%)				75.69		<0.001
No	136 (85.5)	34 (33.0)	174 (63.3)			
Yes	23 (14.5)	69 (67.0)	101 (36.7)			
Education, *N* (%)				8.10		<0.05
Primary education	1 (0.6)	0 (0.0)	1 (0.4)			
Secondary education	16 (10.1)	4 (3.9)	20 (7.6)			
Secondary vocational education	64 (40.3)	32 (31.1)	96 (36.6)			
Tertiary education (bachelor, master and/or specialization)	78 (49.1)	67 (65.0)	145 (55.3)			
Living Situation, *N* (%)				20.71		<0.001
Alone	12 (7.5)	22 (21.4)	34 (13.0)			
With parents/family	47 (29.6)	13 (12.6)	60 (22.9)			
In a student house/With friends	7 (4.4)	1 (1.0)	8 (3.1)			
With partner and no children	48 (30.2)	35 (34.0)	83 (31.7)			
With partner and children	41 (25.8)	27 (26.2)	68 (26.0)			
Other living situations	4 (2.5)	5 (4.9)	9 (3.4)			

SD, standard deviation.

### 3.2 Differences in the continuous variables between countries

Table 2 reports countries’ differences regarding T1D-specific data, diabetes distress, emotion regulation strategies (i.e., cognitive reappraisal, expressive suppression, and mood repair), and emotional self-awareness dimensions (i.e., attention to feelings, and clarity of feelings). Overall, the sample showed values above the cut-off for both diabetes distress and glucose control. In particular, *n* = 124 (47.33%) adults with T1D present higher values in glucose control, and *n* = 148 (56.49%) experience elevated diabetes distress. Moreover, Italian and Dutch adults differed significantly in attention to feelings and mood repair, with Dutch adults showing higher values in both variables, though in both cases the effect size is small (*d* = 0.30, *d* = 0.39, respectively).

**Table 2:** Independent sample *t*-test for assessing countries differences.

Variables assessed	Italy	The Netherlands	Total Sample	*t* (df)	*p*
	*n* = 159 Mean (SD)	*n* = 116 Mean (SD)	*N* = 262 Mean (SD)		
Diabetes duration	18.89 (12.95)	19.36 (12.92)	19.08 (12.91)	−0.29 (259)	0.78
Glucose control (%), at time of data collection	7.06 (1.26)	7.34 (1.36)	7.15 (1.30)	−1.63 (246)	0.10
<7%	6.33 (0.39)	6.24 (0.52)	6.3 (0.43)		
≥7%	7.93 (1.40)	8.12 (1.23)	8.01 (1.33)		
Diabetes-related distress (assessed through PAID-5)	8.60 (4.87)	7.98 (4.31)	8.36 (4.66)	1.06 (260)	0.29
<8	3.73 (2.43)	4.32 (2.08)	3.99 (2.29)		
≥8	11.88 (2.99)	11.43 (2.71)	11.72 (2.89)		
Cognitive reappraisal (assessed through ERQ)	4.35 (1.36)	4.47 (1.02)	4.40 (1.24)	−0.76 (260)	0.45
Expressive suppression (assessed through ERQ)	3.34 (1.36)	3.40 (1.21)	3.36 (1.30)	−0.35 (260)	0.73
Mood repair (assessed through TMMS)	19.79 (5.25)	21.71 (4.66)	20.54 (5.10)	−3.03 (260)	<0.05
Clarity of feelings (assessed through TMMS)	36.72 (7.73)	36.38 (8.86)	36.58 (8.18)	0.33 (260)	0.74
Attention to feelings (assessed through TMMS)	48.80 (7.69)	51.11 (7.77)	49.75 (7.76)	−2.29 (260)	<0.05

SD, standard deviation; PAID-5, The Problem Areas in Diabetes Scale-Short Form – 5; ERQ, The Emotion Regulation Questionnaire; TMMS, The Trait-Meta Mood Scale.

Partial Pearson’s r correlation coefficients, evaluated separately in both countries, are reported in Supplementary Table S1.

### 3.3 Multiple linear regression analyses

**Figure 1:**
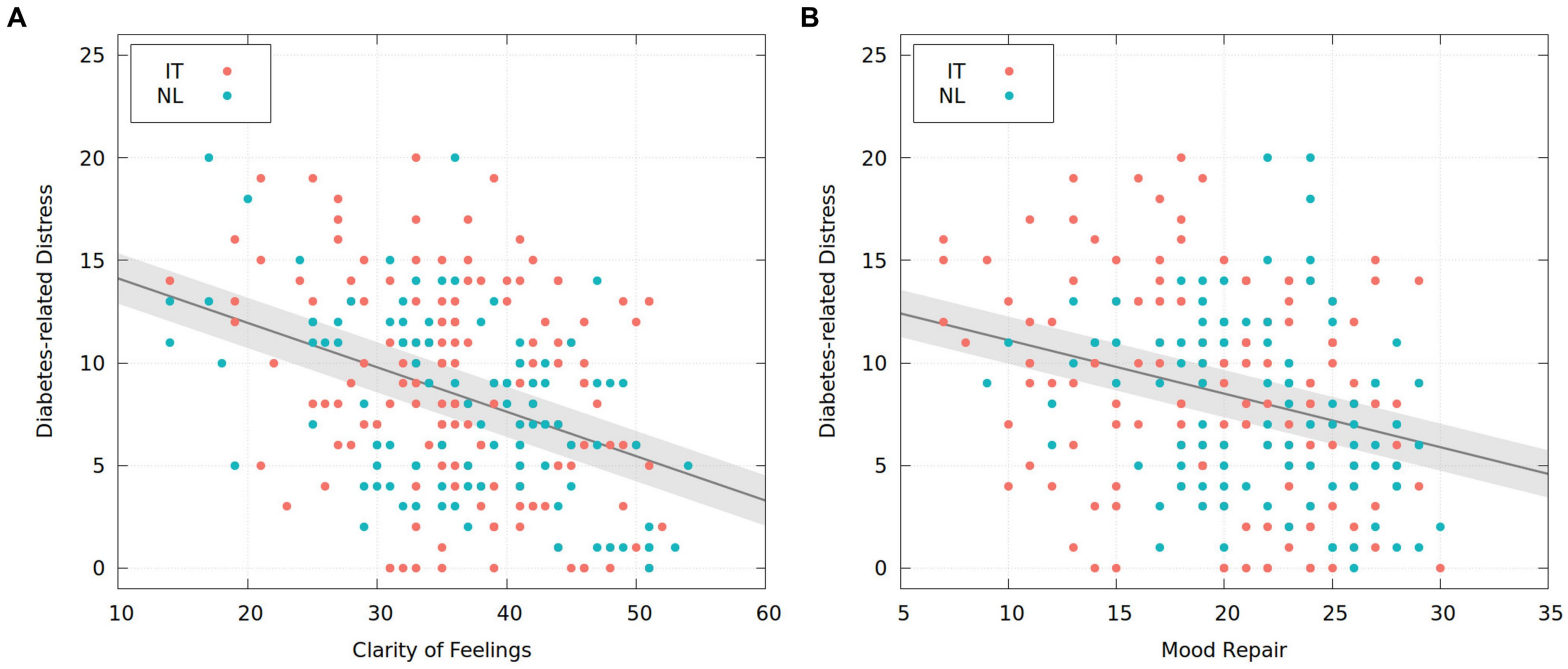
Two multiple regression analyses: the relationship between emotional self-awareness and emotional regulation in type 1 diabetes. IT = Italy; NL = The Netherlands.

The first multiple linear model (see Figure 1A), comprising the two emotional self-awareness dimensions, showed that only clarity of feelings was a statistically significant predictor of diabetes distress [*F*(1,260) = 43.82, *β* = −0.22, *t* = −6.62, *p* < 0.001, *f*^2^ = 0.16], reporting an *R*^2^ = 0.14 of its total variance. The second multiple linear model (see Figure 1B), including the three emotion regulation strategies, revealed that only mood repair was a statistically predictor of diabetes distress [*F*(1,260) = 19.69, *β* = −0.26, *t* = −4.80, *p* < 0.001, *f*^2^ = 0.09], accounting for *R*^2^ = 0.08 of its total variance. In the third and last multiple linear model, the interaction effect between clarity of feelings and mood repair was not significant [*F*(1,257) = 12.33, *β* = −0.00, *t* = −0.36, *p* = 0.72].

## 4 Discussion

Studies have mainly focused on the association between emotion regulation and diabetes distress among individuals with T1D or a combination of people with T1D and T2D (Fisher et al., 2018; Coccaro et al., 2021). The current study extends this literature by providing evidence of a further construct, namely emotional self-awareness, as a potential factor in decreasing diabetes distress among Italian and Dutch adults with T1D. Specifically, we were interested in investigating whether emotional self-awareness could explain a higher proportion of variance compared to emotion regulation.

Interestingly, we found statistically significant differences between the Italian and Dutch adults on a number of variables that lead us to control for the country, in order to pursue the above-mentioned objective. In this regard, concerning emotion regulation strategies and considering the whole sample, only mood repair negatively influenced diabetes distress, accounting for 8% of the total variance and thus resulting in a small effect size. More specifically, the more adults with T1D adopted the mood repair regulatory strategy, the less they experienced diabetes distress. These findings are consistent with the outlined, albeit limited, literature (Fisher et al., 2018; Coccaro et al., 2021). Adaptive emotion regulation strategies are necessary for successful adaptation in a variety of contexts, including affective functioning and social relationships (Gross, 2001; Gross and Thompson, 2007).

Regarding the dimensions of emotional self-awareness, which have not been so far investigated in previous studies, only clarity of feelings negatively predicted diabetes distress, explaining 14% of its variance and thus resulting in a medium effect size. The more adults with T1D were capable of identifying their emotional states and discriminating between them, the less diabetes distress they experienced. In line with this, and interestingly, in both countries, as the clarity of feelings increased, the use of the mood repair regulatory strategy increased and vice versa, showing a medium effect size.

These results may suggest that both constructs can help individuals with T1D to effectively cope with such psychological distress; however, clarity of emotional experience explains a greater proportion of the variance compared to the regulatory strategy of repairing emotions. This can lend support to the hypothesis that individuals initially identify their own emotional states, discern them, and subsequently implement strategies to modulate them (Bagby and Taylor, 1997; Mayer and Salovey, 1997; Rieffe et al., 2008). Indeed, emotional self-awareness plays an important role in the process of regulating emotions, by allowing the individual to modify, accept, and tolerate emotions (Gratz and Roemer, 2004; Berking et al., 2008; Lambie, 2008). In this regard, the non-interacting effect between clarity of feelings and mood repair provides insight into how these two constructs act together, but separately, in reducing diabetes distress. Indeed, from a clinical perspective, the healthcare professionals should act on both in parallel to support the individual in reducing T1D-related distress.

It would seem worthwhile to conduct future studies aimed at investigating the same constructs to consider the same theoretical framework, thus relying on the EPM of emotion regulation (Gross, 2015) and embracing the emotional intelligence-related definition of emotional self-awareness (Bagby and Taylor, 1997; Mayer and Salovey, 1997; Rieffe et al., 2008). When applying the above-mentioned models, it is worth conducting the research in samples of patients mainly composed of adults who experience diabetes distress. Moreover, within this study, we employed the two subscales of the TMMS-30 to assess the emotional self-awareness construct, encompassing clarity of feelings and attention to feelings. Simultaneously, we used the third subscale, that is mood repair to examine the emotional regulation construct, which is also an integral component of emotional intelligence. However, subsequent research would benefit from adopting a unidimensional measure focused solely on exploring emotional self-awareness.

Given the importance of clarity of feelings and mood repair in reducing diabetes distress, psychological interventions could be more beneficial if directed at acquiring or increasing awareness of one’s emotions and developing adaptive emotion regulation strategies to cope with the emotional challenges of living with and managing T1D. It would also be interesting to include the construct of mentalization in the development of psychological interventions for adults with T1D. Mentalization refers to the ability to understand one’s (and others’) experience of one’s own (and others’) mental state, particularly in relation to intense emotional issues, such as the management of T1D, also influencing emotion regulation processes (Fonagy and Allison, 2012). Furthermore, mentalization is related to the individual’s ability to reflect or mentalize the bodily self and that of others (Luyten and Fonagy, 2016). For example, recent studies have shown that mentalization-based interventions can reduce diabetes distress and promote psychological well-being, although so far only among adolescents with T1D (Costa-Cordella et al., 2021; Garrett et al., 2021). This could prevent adults with T1D from adopting maladaptive and ineffective behaviors to cope with the burdens of managing T1D.

The present study provides useful evidence for research and clinical practice regarding the association between dimensions of emotional self-awareness, emotion regulation strategies, and T1D-related distress. However, the results of the current study should be interpreted considering some limitations, which can be addressed in future studies. First, this is a cross-sectional study; nevertheless, it has been useful in identifying variables relevant to clinical practice and their investigation in future large longitudinal studies. Longitudinal studies should aim to understand in depth the role of these variables and their consolidated link, by considering both protective factors, in adults with T1D experiencing diabetes distress. Moreover, a notable gender imbalance existed within our sample, with females representing 80.5%, which constrains reliable gender-based analytical outcomes. In addition, outcomes were self-reported, hence participants’ responses could be biased; future studies could include other assessment measures, such as *ad hoc* semi-structured interviews, in order to investigate from a qualitative perspective how adults identify, understand, and discern their emotional states and how they, in turn, regulate them, by further exploring which emotional regulation strategies they adopt when faced with different situations, particularly in managing T1D. In this regard, it is important to be mindful that individuals should have a deep understanding of the emotional regulation strategy to be implemented in order to identify the appropriate one for the specific situation (Gross, 2015).

Overall, gaining a deeper understanding of the emotional factors that enable a decrease in symptoms related to diabetes distress could contribute to the improvement of psychological interventions and outcomes for adults with T1D.

## Data availability statement

The raw data supporting the conclusions of this article will be made available by the authors, without undue reservation.

## Ethics statement

The studies involving humans were approved by both the ethics committee of the Amsterdam University Medical Centres-location VUmc (2021.0452) and the University of Padua, Italy (2021.4247). The studies were conducted in accordance with the local legislation and institutional requirements. The participants provided their written informed consent to participate in this study.

## Author contributions

GB: Conceptualization, Data curation, Formal Analysis, Investigation, Methodology, Project administration, Writing – original draft. JE: Investigation, Writing – review & editing. MW: Supervision, Writing – review & editing. FS: Supervision, Writing – review & editing. SS: Supervision, Writing – review & editing.
