# Supplementary material for: The relationship between emotional self-awareness, emotion regulation, and diabetes distress among Italian and Dutch adults with type 1 diabetes
Source: Front Psychol. 2023 Nov 22;14:1288550. doi: 10.3389/fpsyg.2023.1288550 (PMC10702723; doi:10.3389/fpsyg.2023.1288550)
Supplement: Supplementary file 1 [file Table_1.DOCX]

Supplementary Material

**Table S1.** Pearson’s partial correlations among adults with DT1 in Italy vs. in The Netherlands

|  | **1.** | **2.** | **3.** | **4.** | **5.** | **6.** | **7.** | **8.** |  |
| --- | --- | --- | --- | --- | --- | --- | --- | --- | --- |
| **1. Age** |  | -0.15 | -0.15 | -0.02 | **-0.27*** | 0.01 | **0.28**** | 0.19 |  |
| **2. Glucose control (%)** | -0.01 |  | **0.22*** | -0.12 | -0.12 | -0.13 | -0.20 | -0.04 |  |
| **3. Diabetes-related Distress** | 0.11 | 0.03 |  | -0.17 | 0.09 | -0.07 | **-0.48**** | -0.21 |  |
| **4. Cognitive Reappraisal** | 0.01 | 0.02 | **-0.21**** |  | -0.12 | **0.34**** | **0.42**** | **0.54**** |  |
| **5. Expressive Suppression** | -0.11 | 0.02 | 0.08 | **0.32**** |  | **-0.37**** | **-0.49**** | **-0.25*** |  |
| **6. Attention to Feelings** | 0.02 | -0.05 | -0.03 | 0.08 | **-0.41**** |  | **0.21*** | 0.15 |  |
| **7. Clarity of Feelings** | 0.17 | -0.05 | **-0.26**** | **0.28**** | **-0.33**** | **0.39**** |  | **0.45**** |  |
| **8. Mood Repair** | 0.12 | **0.19*** | **-0.25**** | **0.41**** | **-0.14**** | **0.24**** | **0.46**** |  |  |
| *Note.* Below the diagonal are reported the correlations for Italy; above the diagonal are reported the correlations for The Netherlands; N = 262; **p<0.01; *p<0.05 | | | | | | | | | |
